# High innate preference of black substrate in the chive gnat, *Bradysia odoriphaga* (Diptera: Sciaridae)

**DOI:** 10.1371/journal.pone.0210379

**Published:** 2019-05-09

**Authors:** Lina An, Xiaofan Yang, Klaus Lunau, Fan Fan, Mengyao Li, Guoshu Wei

**Affiliations:** 1 College of Plant Protection, Hebei Agricultural University, Baoding, China; 2 Institute of Sensory Ecology, Biology Department, Heinrich-Heine-University Düsseldorf, Düsseldorf, Germany; Chinese Academy of Agricultural Sciences, CHINA

## Abstract

The chive gnat, *Bradysia odoriphaga*, is a notorious pest of *Allium* species in China. Colour trapping is an established method for monitoring and control of *Bradysia* species. In order to clarify the effect of colour preference of *B*. *odoriphaga* for the perched substrate, multiple-choice tests were used to assess the response of the chive gnat to different colour hues and brightness levels under different intensities of white illumination and two spectrally different illuminations. Given the choice among four colours differing in hue under different intensities of white illumination and two spectrally different illuminations, chive gnat adults significant preferred the black substrate, a lesser preference to brown and green substrates, and the least preference to orange substrate irrespective of illumination. Given the choice among four levels of brightness under the same illumination conditions as those in the previous experiment (different intensities of white illumination and two spectrally different illuminations), chive gnats preferred black substrate over dark grey, light grey and white substrates. Meanwhile, both virgin and mated adults significantly preferred black over other colour hues and brightness. Based on our results, we conclude that the chive gnat adults significantly prefer black substrates irrespective of colour hues and brightness. This behaviour does not alter with ambient light condition changes. No difference observed between choices of female and male adults. Our results provide new insight for understanding the colour choice behaviour in chive gnat and pave a way to improve monitoring and control of chive gnats and management.

## Introduction

The chive gnat, *Bradysia odoriphaga* (Diptera: Sciaridae), is the most destructive pest to *Allium* vegetables in China, especially to Chinese chive *Allium tuberosum*. Although chive gnat adults do not cause plant damage, the females lay eggs around the root in soil, where larvae hatched directly damage roots and bulbs of plants, thus disrupting the uptake of water and nutrients[1]. Historically, the control of chive gnat has seriously dependent on the use of chemicals, such as chlorpyrifos and phoxim[2–3], but the control effect is limited mainly due to the cryptic larval life style and the development of resistance to insecticides[4]. Particularly, over use of certain pesticides may lead to environmental pollution and high pesticide residues. Therefore, it is necessary and exigent to search safe and efficient management strategies to control chive gnat.

Many insects use visual stimuli to perceive a variety of resources, e.g. adult food, mating encounter sites, oviposition sites or shelter from harmful biotic or abiotic conditions [5–6]. The perception quality of visual objects, however, is strongly influenced by the characteristics of reflected light including hue and brightness. Colour is especially important for distinguishing resource quality, e.g. flower condition, partner selection [7–10], as well as location (e.g. oviposition site, shelter) [11–12]. In this study we investigated the preference of chive gnat for colour hues and brightness of chive gnat in order to reduce the damage of *A*. *tuberosum* by means of monitoring or controlling the chive gnat.

Vision-orientated coloured sticky traps may represent relevant potential monitoring and control strategies of *Bradysia* spp. [13], since these trapping methods are environment-friendly and do not cause pesticide residues and pesticide resistance. Colour trapping is a common method for trapping various insect species [14]. Many insects have already been confirmed to exhibit colour preferences including those for distinct colour hues, colour saturation, colour brightness, and colour contrast [15–17]. Most profound studies about innate colour preferences in insects focused on pollinating insects such as bees [10,18–19], lepidopterans [20–21], and flies [9,22–23], whereas studies about colour preferences in agricultural pests mostly evaluated the results of colour trapping [24–25]. Colour trapping is also a common method for the control of dipteran pest species. For example, the whitefly, *Bemisia tabaci*, some tephritid flies, and anthomyid flies, *Strobilomyia* spp. are particularly attracted by yellow sticky traps [26–28], whereas the blue blowfly, Calliphora vomitoria, exhibits an innate colour preference for black [29] and fungus gnat, *Bradysia difformis*, significant preferred black surface rather than yellow LEDs [30]. Actually, in field experiments with coloured sticky traps chive gnat has already been successfully captured [31], but the quantitative analysis of the contribution of colour parameters such as hue and brightness to lure chive gnats has never been concerned so far.

The purpose of our experiments was to determine the relative attractiveness of different colours to adult chive gnats and to assess the efficacy of colour parameters, hue and brightness, to attract chive gnats. In addition we investigated whether the chive gnat adults maintained their innate colour preference when the colour stimuli were presented under various light intensities of white illumination and two spectrally different illuminations. The outcome of these experiments will lead to a better understanding of their colour choice behaviour and colour vision and is thereby beneficial to understand their biological characteristics and develop specific monitoring tools and efficient control strategies.

## Materials and methods

### Insects

*Bradysia odoriphaga* larvae were initially obtained during May 2018 from a field of *Allium tuberosum* located in Cangzhou city of Hebei Province, PR China. The field owner Guangsheng Liu gave permission to collect the chive gnats on this site. The studies did not involve endangered or protected species. The colony of *B*. *odoriphaga* was maintained in the IPM Laboratory of Hebei Agricultural University, Hebei Province, PR China. It was reared on *A*. *tuberosum* for more than 6 generations where eggs, larvae and pupae were mass cultured in Petri dishes (9cm in diameter, 2.5cm height) containing a Whatman No.1 filter paper (soaked with 2.5% agar medium), and the fresh chive *A*. *tuberosum* was placed in a separate petri dish as diet for the larvae. The chive gnat adults were placed in rearing containers made of plastic pots (9cm top diameter, 15cm bottom diameter, 5cm height). A petri dish (15cm diameter) was used as the bottom of each rearing container, and a reversed plastic cup (9cm top diameter, 15cm bottom diameter, 5cm height; with dozens of needle holes for gas exchange) was used as the cover. The container between the bottom and the cover was sealed with sealing film. Each petri dish contained a filter paper that was soaked with 2.5% agar medium (about 15ml) for maintaining moisture. Newly emerged female and male gnats could mate immediately while emale gnats can lay eggs one or two days after mating. Insect colonies were maintained in climate chambers maintained at 24±1°C with 75±5% relative humidity and a L14:D10 hours photoperiod.

### Colour hue and brightness

**Fig 1:**
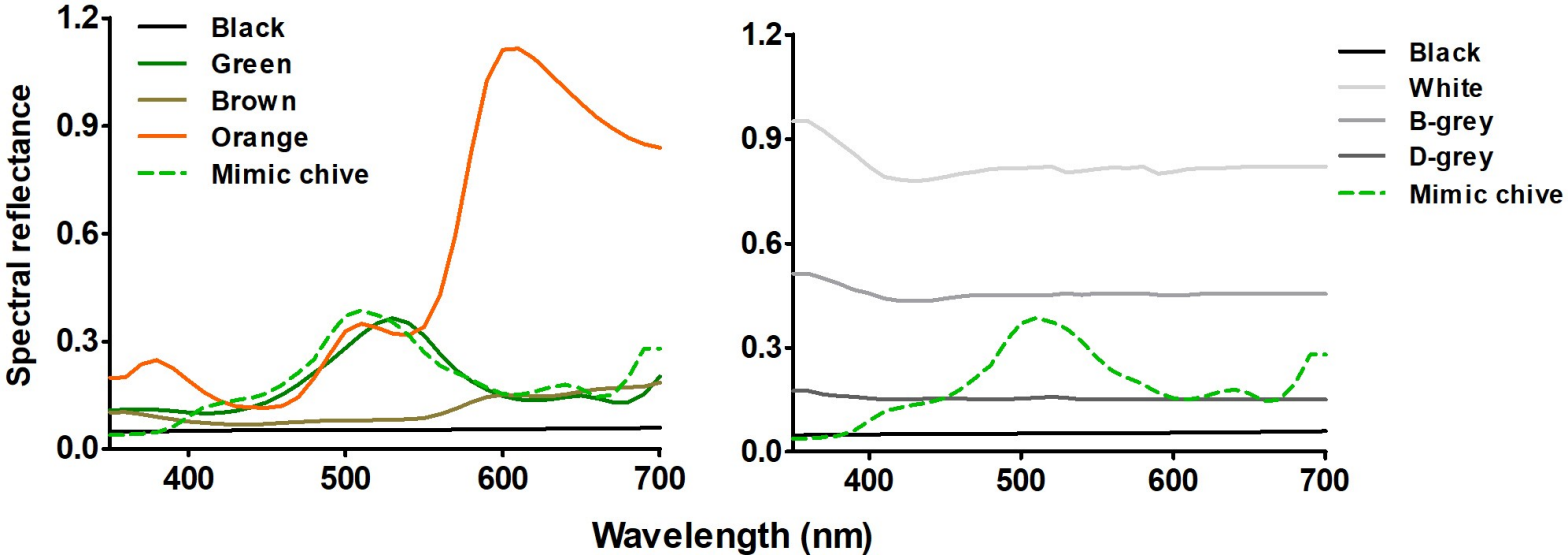
Reflectance spectra of all colour stimuli used in the experiments. The stimuli of colour hues were on the left; the stimuli of brightness were on the right). L-grey means light grey, D-grey means dark grey.

Based on the colours of adult body surface, the fresh and old host plants, and of the soil green, orange, brown, and black colour papers were selected as stimuli for the experiment with varying colour hues. Four brightness levels including white, light grey, dark grey, and black were selected for the experiment with varying colour brightness. Colour papers made of photographic paper printed via POWERPOINT printed by a colour inkjet printer (HP 100) were offered (Table 1). The spectral reflectance of the colour stimuli was measured by a spectrophotometer (Konica Minolta CM-3700A, Japan) (Fig 1). Light emitting diodes (LEDs) used in experiment were designed with specific ranges of wavelengths, such as 525 to 530nm for green light, 455 to 460nm for blue light and white light with a colour temperature of 6000~6500K. All intensities of LEDs were measured by illuminometer (TES-1339, Tes Electronics Industry Corporation, PR China).

**Table 1:** The RGB values of the different colour hues and brightness levels.

Experiment	Colour stimuli	RGB value
Colour hues	Black	0, 0, 0
	Brown	120, 60, 0
	Green	0, 145, 65
	Orange	245, 140, 0
Brightness levels	Black	0, 0, 0
	White	255, 255, 255
	L-grey	190, 190, 190
	D-grey	90, 90, 90

### Experimental device

**Fig 2:**
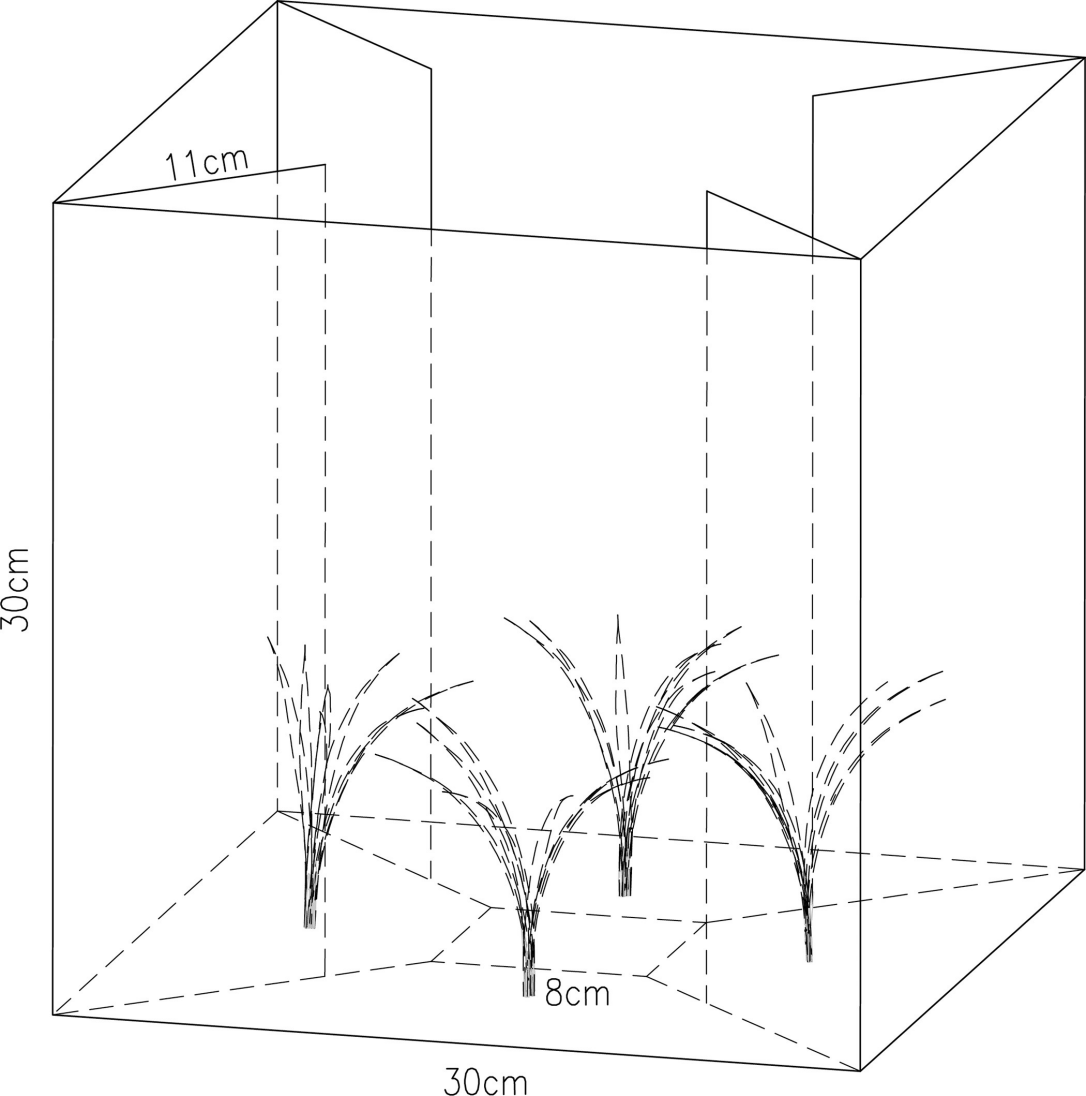
Device of the colour choice tests with *Bradysia odoriphaga*. Scheme of the device of colour choice test, which was designed as a cube (edge length = 30cm). In the middle of cube was small square (edge length = 8cm) used as a decision area. The chambers were separated by cardboard. All four differently coloured chambers contained a mimic chive plant.

The device used for multiple choice tests was a quadrilateral cube (length×width×height = 30×30×30 cm^3^) made up of cardboards and has four chambers of identical size. In the middle of the device was the release zone displaying a white colour (length×width = 8×8 cm^2^) of flies to be tested (Fig 2). Bottom of each chamber in the middle contained an artificial Chinese chive. The device has a lid made of Plexiglas which was used to prevent chive gnat adults from flying out of the device (Fig 2).

## Experimental procedure

Based on the activity characteristics of chive gnat adults [32], all the experiments were started at 9:00 am every day. The experimental conditions simulated those of greenhouses used for growing Chinese chive due to chive gnats were tested in behavioural device with a transparent lid. Before the tests the chive gnat adults were placed in a completely dark environment for 30 min for equal adaptation. In the experiments of innate preference for colour hues and brightness levels, each chamber was pasted with one of four coloured papers to be used as a colour chamber for quadruple choice.

### Experiment 1: Colour hue and brightness preference under four intensities of white illumination

The rationale of the experiments was to study the innate preference of chive gnat adults to respond different colour hues and brightness of stimuli. Four colours differing in hues (black, orange, brown and green) were used to test the colour preference of chive gnats under four different intensities of white illumination, respectivelyIn addition, four brightness levels of stimuli (white, light grey, dark grey and black) were used to test the brightness preference of chive gnat under four different intensities of white illumination, respectively. For each trial 30 newly emerged and healthy adults were put into the release zone of the device under white illumination with 0.1, 100, 1000, 10000lux, respectively. And the device was immediately covered with a transparent lid. After 30 min the number of flies in each chamber was counted. Each treatment was repeated 20 times. Ten trials were performed each with females and males separately.

### Experiment 2: Colour hues and brightness discrimination under two spectrally different illuminations

The rationale of the experiments was to study whether chive gnat adults can maintain the colour preference when tested under spectrally different illuminations. The same colour stimuli, four colour hues (black, grren, brown and orange) and four brightness (white, light grey, dark grey and black), were respectively used to test the colour choice of chive gnats under blue or green illumination with 250lux. For each trial 30 newly emerged and healthy adults were put into the release zone of the device under blue and green light with 250lux, respectively. And the device was immediately covered with a transparent lid. After 30 min the number of flies in each chamber was counted. Each treatment was repeated 20 times. Ten trials were performed each with females and males separately.

### Experiment 3: Colour hues and brightness of chive gnat adults with different physiological state

The rationale of the experiments was to study the innate preference of virgin and mated adults to respond different colour hues and brightness of stimuli. Newly emerged female adults (or male adults) were placed in a new rearing container before mating, and were used as virgin adults for testing colour preference. However, for mated adults, we put newly emerged female and male adults into one rearing container, then they were separated after mating, and were used as mated adults for testing colour preference. For each trial, virgin adults and mated adults were put into the release zone of the device under white illumination with 100 lux, respectively. And the device was immediately covered with a transparent lid. After 30min the number of flies in each chamber was counted. Each treatment was repeated 20 times. Ten trials were performed each with virgin and mated females separately and 10 trials were performed each with virgin and mated males sparately.

## Results

### Experiment 1: Colour hues and brightness preference under four intensities of white illumination

**Fig 3:**
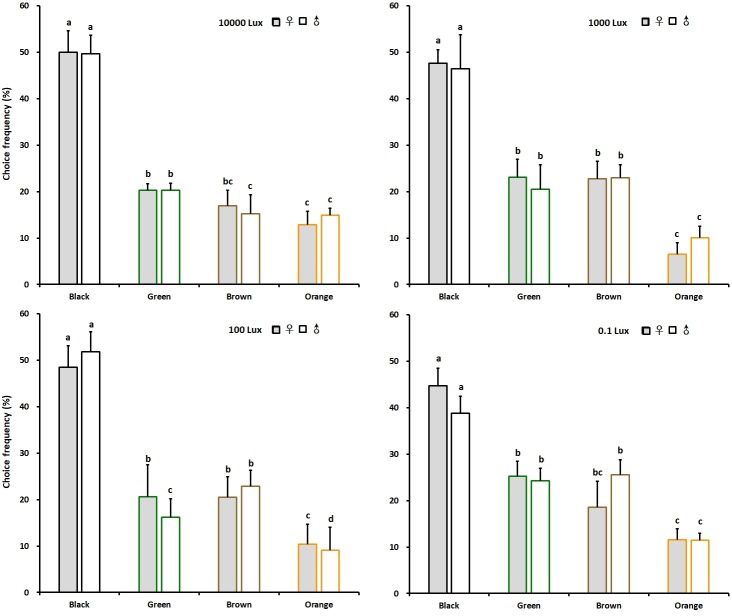
Colour choices in chive gnat *Bradysia odoriphaga* adults of different colour hues under four different intensity of white illumination. Different letters refer to significant differences according to One-way ANOVA with *P*<0.05.

In the colour preference experiments, both the female and male adults significantly preferred the black colour irrespective of light intensities while other colours (i.e. green, brown and orange) were less attractive (Fig 3). Female and male adults visited the black with a choice frequency of 50.00 and 49.63% for 10000 lux, 47.64 and 46.45% for 1000 lux, 48.47 and 51.85% for 100 lux and 44.68 and 38.75% for 0.1 lux intensity. The brown and green colours were significantly less attractive. The orange colour was seemingly the least attractive, the choice frequencies of female and male were 12.84 and 14.85% for 10000 lux, 6.50 and 10.09% for 1000 lux, 10.41and 9.09% for 100 lux and 11.57 and 11.44% for 0.1 lux intensity.

**Fig 4:**
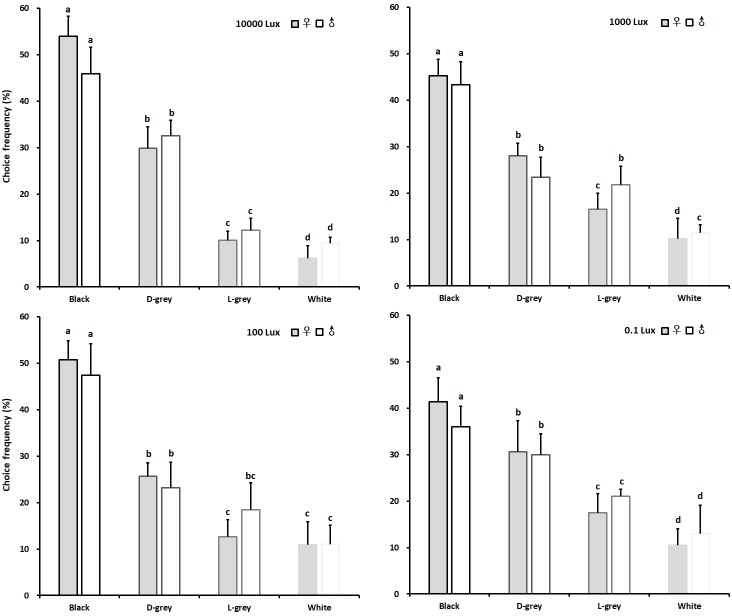
Colour choices of stimuli varying in brightness in chive gnat *Bradysia odoriphaga* adults under four different intensities of white illumination. Different letters refer to significant differences according to One-way ANOVA with *P*<0.05.

In the brightness choice experiments, both the female and male adults also significantly preferred the black colour irrespective of light intensities, while other brightness levels were less attractive (Fig 4). Female and male adults visited the black with a choice frequency of 53.92 and 45.87% for 10000 lux, 45.29 and 43.33% for 1000 lux, 50.78 and 47.38% for 100 lux and 41.41 and 36.00% for 0.1 lux intensity, respectively. The choice frequencies of female and male adults for dark grey were 29.84 and 32.54% for 10000 lux, 28.05 and 23.43% for 1000 lux, 25.63 and 18.42% for 100 lux and 30.66 and 30.00% for 0.1 lux intensity. The light grey and white colour were less attractive. The control experiment using the device with the same colour (white colour) in each chamber under 100 lux white illumination showed that chive gnats did not prefer one of the chambers (S1 Fig). The colour preference was similar for males and females (Table 2).

**Table 2:** Variance analysis of the response of *Bradysia odoriphaga* to colour hues and brightness under different light intensities and wavelengths.

Experiment	Influencing factors	*df*	Mean square	*F*	Sig.
exp.1 Colour hues and Brightness	Colour hues	3	19423.436	1135.185	0.000
	Sex	1	2.531E-005	0.000	0.999
	Light intensity	3	4.479E-006	0.000	1.000
	Colour hues×Light intensity	9	244.133	14.268	0.000
	Colour hues×Sex	3	69.619	4.069	0.007
	Light intensity×Sex	3	2.813E-006	0.000	1.000
	Colour hues×Light intensity×Sex	9	65.576	3.833	0.000
	Brightness	3	19020.912	871.172	0.000
	Sex	1	0.959	0.044	0.834
	Light intensity	3	1.282	0.059	0.981
	Brightness×Light intensity	9	382.734	17.530	0.000
	Brightness×Sex	3	263.574	12.072	0.000
	Light intensity×Sex	3	1.294	0.059	0.981
	Brightness×Light intensity×Sex	9	42.232	1.934	0.047
exp.2 Colour hues and Brightness	Colour hues	3	8479.539	595.810	0.000
	Sex	1	6.250E-007	0.000	1.000
	Wavelength	1	1.563E-005	0.000	0.999
	Colour hues×Sex	3	51.017	4.054	0.008
	Colour hues×Wavelength	3	59.165	4.702	0.004
	Wavelength×Sex	1	0.000	0.000	0.998
	Colour hues×Sex×Wavelength	3	57.878	4.599	0.004
	Brightness	3	8978.405	764.689	0.000
	Sex	1	5.625E-006	0.000	0.999
	Wavelength	1	3.063E-005	0.000	0.999
	Brightness×Sex	3	39.4635	3.361	0.021
	Brightness×Wavelength	3	72.919	6.210	0.001
	Wavelength×Sex	1	5.625E-006	0.000	0.999
	Brightness×Sex×Wavelength	3	463.315	39.460	0.000
exp.3 Colour hues and Brightness	Colour hues	3	10883.918	838.436	0.000
	Sex	1	5.635E-006	0.000	0.999
	Physiological state	1	5.635E-006	0.000	0.999
	Colour hues×Sex	3	78.115	6.018	0.001
	Colour hues×Physiological state	3	6.599	0.508	0.677
	Physiological state×Sex	1	6.250E-007	0.000	1.000
	Colour hues×Sex×Physiological state	3	21.422	1.650	0.180
	Brightness	3	10955.784	1988.498	0.000
	Sex	1	4.000E-005	0.000	0.998
	Physiological state	1	2.500E-006	0.000	0.999
	Brightness×Sex	3	22.902	4.157	0.007
	Brightness×Physiological state	3	10.816	1.963	0.122
	Physiological state×Sex	1	0.000	0.000	1.000
	Brightness×Sex×Physiological state	3	12.631	2.293	0.081

Note: All the data were analyzed by Multifactor Variance Analysis (SPSS 17.0)

### Experiment 2: Colour hues and brightness discrimination under two spectrally different illuminations

**Fig 5:**
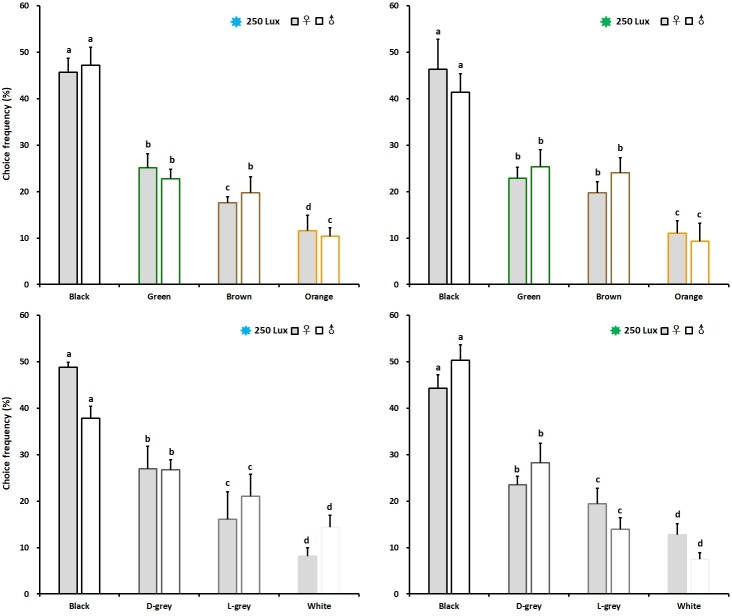
Colour hues and brightness preference of chive gnat *Bradysia odoriphaga* adults under two spectrally different illuminations. Colour choice among four colour stimuli differing in hue under blue light (left) and green light (right) on the top. Choice among four colour stimuli differing in brightness under blue light (left) and green light (right) on the bottom. Different letters refer to significant differences according to One-way ANOVA with *P*<0.05.

Given the multiple choice among four colours differing in hues, black, green, brown and orange, with blue light of 250 lux intensity, the choice frequencies of female and male adults for black were 45.71 and 47.15%, respectivel, which were significantly higher than those for blue, brown and orange colours. The orange colour was seemingly the least attractive, the choice frequencies of female and male were 11.58 and 10.37%. A similar result was obtained testing the chive gnats in green light of 250 lux intensity; 46.31% of female adults and 41.33% male adults both significantly preferred the black than other three colours. The orange colour was seemingly the least attractive, the choice frequencies of female and male were 11.08 and 9.33% (Fig 5 Up). Moreover, the multiple choice among four levels of brightness (black, dark grey, light grey and white) under blue light of 250 lux intensity were used for testing the choice preference of the chive gnat adults. The adult females significantly preferred black with a percentage of choice amounting to 48.82% over dark grey amounting to 26.97% as well as light grey and white amounting to 16.10 and 8.12%, respectively. the adult males, however, significantly preferred black with a percentage of choice amounting to 37.79% over dark grey amounting to 26.76% as well as light grey and white amounting to 21.07 and 14.38%. Under green light of 250 lux intensity the adult females significantly preferred black with a choice frequency amounting to 44.32% over dark grey, light grey and white amounting to 23.47, 19.47 and 12.74%, respectively. However, the male adults significantly preferred black with a choice frequency amounting to 50.31% over dark grey, light grey and white amounting to 28.22, 13.97 and 7.50%, respectively (Fig 5 Down).

### Experiment 3: Colour hues and brightness of chive gnat adults with different physiological state

**Fig 6:**
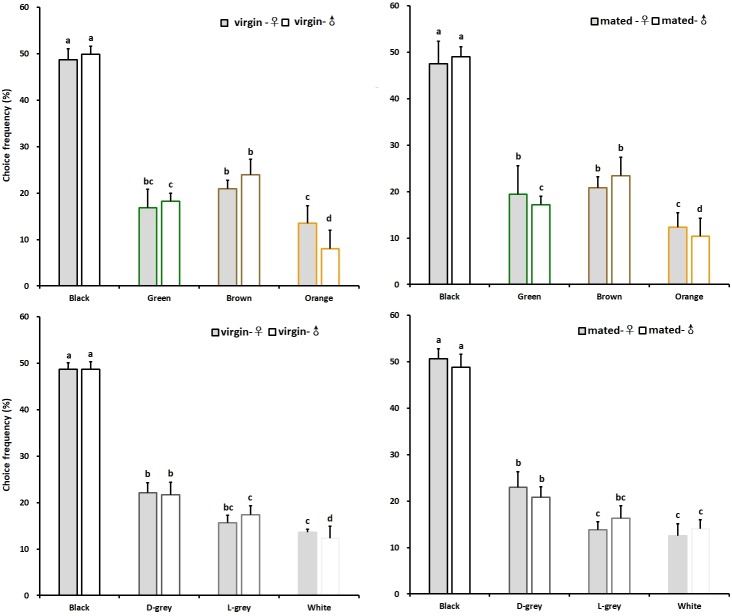
Colour and brightness preference of chive gnat *Bradysia odoriphaga* adults with different physiological states. Different letters refer to significant differences according to One-way ANOVA with *P*<0.05.

In the colour choice experiments both the virgin female and male adults significantly preferred the black colour, with a choice frequency amounting to 48.67 and 49.85%, respectively. To a lesser preference to brown and green, whereas orange was significantly less attractive, the choice frequencies of mated females and males were 13.52 and 8.06%, respectively (Fig 6 Left). However, the mated female and male adults both significantly preferred the black colour with a choice frequency amounting to 47.46 and 49.05%, respectively, to a lesser preference to brown and green, and orange was significantly less attractive, the choice frequencies of mated females and males were 12.30 and 10.38%, respectively (Fig 6 Right). By contrast, in the brightness experiment, both the virgin female and male adults significantly preferred the black colour with a choice frequency amounting to 48.66 and 48.67%, respectively, which was significantly higher than dark grey, light grey and white, and white was significantly less attractive, the choice frequencies of mated females and males were 13.63 and 12.33%, respectively (Fig 6 Left). However, the mated female and male adults also significantly preferred the black colour with a choice frequency amounting to 50.68 and 48.83%, respectively, which was significantly higher than dark grey, light grey and white colours, and white colour was significantly less attractive, the choice frequencies of mated females and males were 12.51 and 14.02%, respectively (Fig 6 Right). In all experiments there was no significant difference in colour choice behaviour between female and male in chive gnat, and also no significant differences in colour preference between virgin and copulated adults were found (Table 2).

## Discussion

Adult chive gnats, *Bradysia odoriphaga*, showed a significant preference for the black substrate, while other coloured substrates attracted only a limited number of chive gnat. These results provide strong evidence that chive gnats possess an innate colour preference for black substrates and some evidences that they maintain the preference for black even if the ambient light conditions change, i.e. the preference for black is not altered by intensity and spectral composition of the illuminating light. Although the tests were specifically designed to capture female chive gnats referring to searching their mates, no differences in the colour preference between the virgin and mated adults of *B*. *odoriphaga* were found and there were no differences in the colour preference between female and male. We, therefore, speculated that chive gnat adults prefer black substrate not only for searching their mates, but also for other reasons, such as searching for oviposition site or finding a safe place for hiding in camouflage due to their black surface. Colour traps have been used to control *Bradysia* gnats under field conditions [31,33], but innate colour preferences have never been investigated in detail for chive gnats, *B*. *odoriphaga*.

Wavelength-specific behaviours known from specific tasks such as oviposition in butterflies (Lepidoptera) and flies (Diptera) regularly are dependent of intensity [34]. Gravid females of certain mosquitos, *Toxorhynchites moctezuma* and *T*. *amboinensis* [11], oviposited preferentially into black substrate. Remarkably, the flower-visiting hoverfly *Eristalis tenax* prefers yellow colours, can learn many other colours, but strongly avoids dark colours [9]. The canopy ant, *Cephalotes atratus*, prefers bright white colours when given a choice of target colours of varying shades of grey, specifically brightness seems to have a great influence on the landing behavior of canopy ants, thus it is suspected that the high contrast between tree trunks and the darker surrounding foliage provides the preferred visual target for falling ant [35].

Likewise, other dipteran pests are known to be attracted to black colours such as the bluebottle fly, *Calliphora vomitoria* [29] and the tabanid fly, *Tabanus illotus* [36]. The preference for black surfaces found in water-living insects [37] and tabanid flies is associated with the perception of horizontally polarized light reflected from shiny surfaces such as water which is optimally seen at black targets [38]. Since the target colours used in the colour choice tests with the chive gnat were not shiny and the light source was not the sun, it is very unlikely that polarization vision might have influenced the colour choice of the chive gnat.

**Fig 7:**
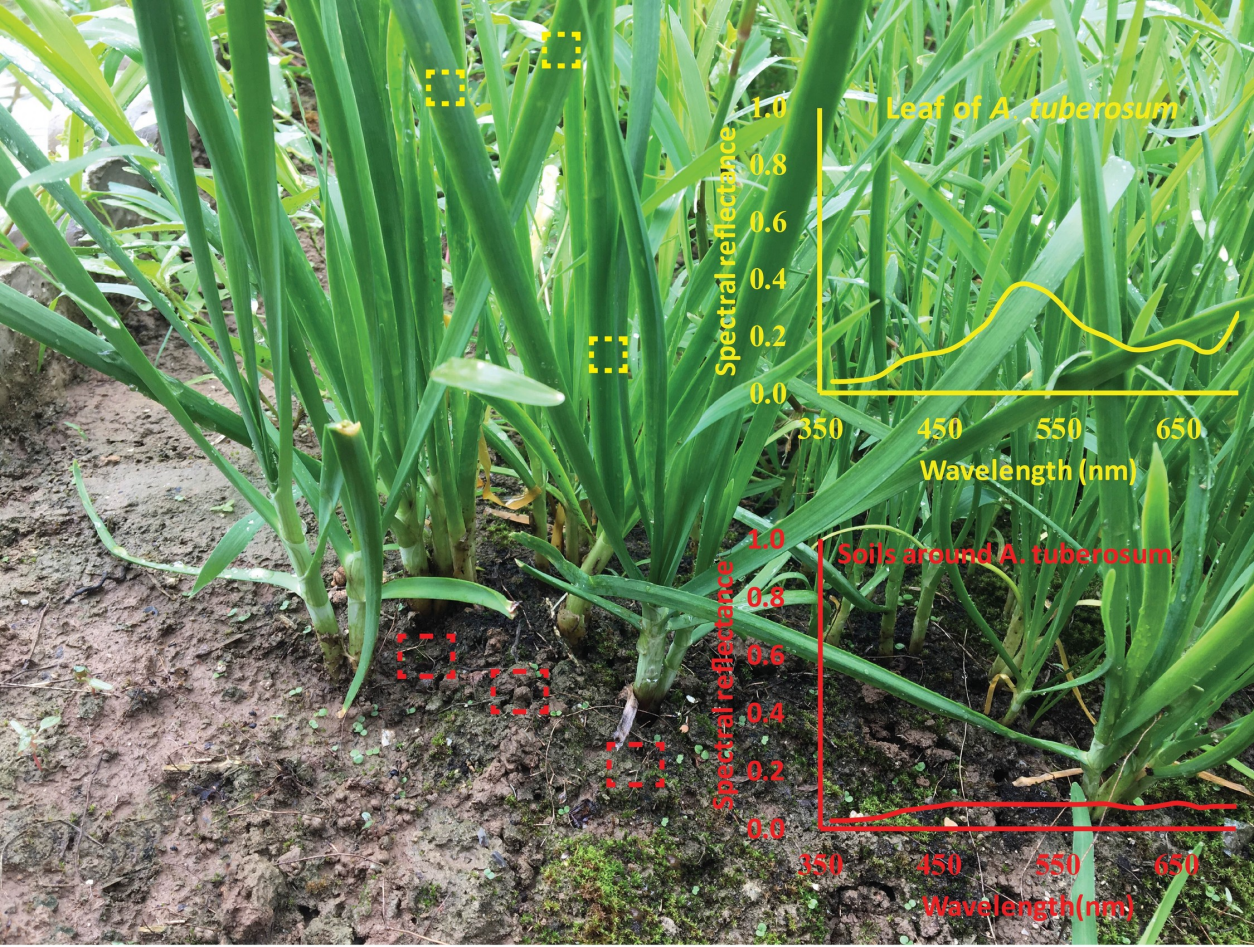
Reflectance spectra of living environment in chive gnat *Bradysia odoriphaga*. Dashed boxes with yellow colour represent the measure sites of leaves of *A*. *tuberosum*. Dashed boxes with red colour represent the measure sites of soils around *A*. *tuberosum*.

The black surface was preferred by chive gnats in comparison to all other colours. One possible reason is that the chive gnats performed a colourblind choice relying only on the contrast between the black target and other colours and the background which is one of the key features for object perception of insects [39] including Diptera [40]. A strong brightness contrast may be found in nature between light plant stems, leaves and the dark substrate. The finding that adult fungus gnats, *B*. *difformis*, significantly preferred black sticky traps over yellow LED, was interpreted that fungus gnat adults were searching for a convenient egg-laying substrate [30]. Based on this hypothesis, the spectral reflectance of the host plant (*Allium tuberosum*) and soil substrate close to root of the chive gnat was measured (Fig 7). The maximum value of spectral reflectance of leaves (*A*. *tuberosum*) is 38%, whereas the maximal reflectance of soil substrate is about 9%, which is very close to the value of the black colour in our experiments (black: 6%). As a conclusion, we assume that the strong colour contrast between the substrate for egg-laying and the host plant might guide the search for mating partners or oviposition sites in dim surroundings.

Although habitat-related olfactory stimuli have been identified as important cues in the specific task of some insects [41–44], visual stimuli should not be overlooked as an important sensory modality, especially the aspect of searching and finding host plant (Reeves, 2011) [45]. In our study, we focused on the effect of visual stimuli for chive gnats, not olfactory stimuli, so a scentless artificial host plant was used as an attractive signal in order to improve the activity of the chive gnat adults. Further studies are needed to explore the interaction of visual and olfactory cues for oviposition and the visual mechanism underlying the colour choice in chive gnats, i.e. photoreceptor types, visual system and their visual ecological significance.

## Supporting information

S1 Fig The randomness test of choice for chive gnat *Bradysia odoriphaga* at 4 chambers with same colour. (DOCX)

S1 Table (XLSX)
