# Supplementary material for: High innate preference of black substrate in the chive gnat, Bradysia odoriphaga (Diptera: Sciaridae)
Source: PLoS One. 2019 May 9;14(5):e0210379. doi: 10.1371/journal.pone.0210379 (PMC6508717; doi:10.1371/journal.pone.0210379)
Supplement: S1 Fig — (DOCX) [file pone.0210379.s001.docx]

**Supplement 1**

**
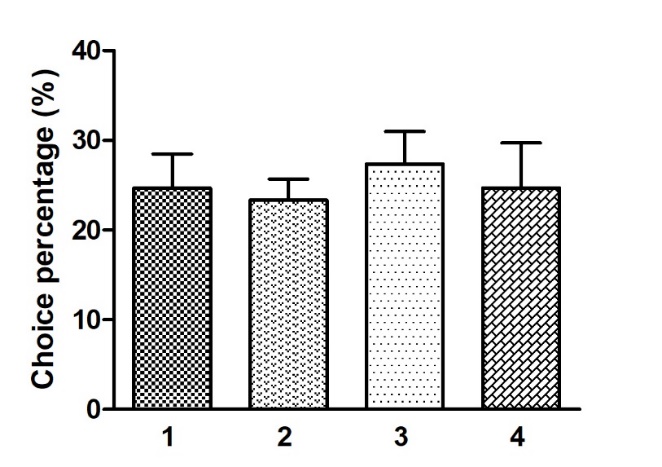
**

Fig. 1 The randomness test of choice for chive gnat *Bradysia odoriphaga* at 4 chambers with same colour
